# Reduction of diabetes-related distress predicts improved depressive symptoms: A secondary analysis of the DIAMOS study

**DOI:** 10.1371/journal.pone.0181218

**Published:** 2017-07-10

**Authors:** André Reimer, Andreas Schmitt, Dominic Ehrmann, Bernhard Kulzer, Norbert Hermanns

**Affiliations:** 1 Research Institute of the Diabetes Academy Mergentheim (FIDAM), Bad Mergentheim, Germany; 2 Diabetes Center Mergentheim (DZM), Bad Mergentheim, Germany; 3 German Center for Diabetes Research (DZD), Munich-Neuherberg, Germany; 4 Otto-Friedrich-University of Bamberg, Department for Psychology, Bamberg, Germany; Institute of Endocrinology and Metabolism, ISLAMIC REPUBLIC OF IRAN

## Abstract

**Objective:**

Depressive symptoms in people with diabetes are associated with increased risk of adverse outcomes. Although successful psychosocial treatment options are available, little is known about factors that facilitate treatment response for depression in diabetes. This prospective study aims to examine the impact of known risk factors on improvement of depressive symptoms with a special interest in the role of diabetes-related distress.

**Methods:**

181 people with diabetes participated in a randomized controlled trial. Diabetes-related distress was assessed using the Problem Areas In Diabetes (PAID) scale; depressive symptoms were assessed using the Center for Epidemiologic Studies Depression (CES-D) scale. Multiple logistic and linear regression analyses were used to assess associations between risk factors for depression (independent variables) and improvement of depressive symptoms (dependent variable). Reliable change indices were established as criteria of meaningful reductions in diabetes distress and depressive symptoms.

**Results:**

A reliable reduction of diabetes-related distress (15.43 points in the PAID) was significantly associated with fourfold increased odds for reliable improvement of depressive symptoms (OR = 4.25, 95% CI: 2.05–8.79; *P*<0.001). This result was corroborated using continuous measures of diabetes distress and depressive symptoms, showing that greater reduction of diabetes-related distress independently predicted greater improvement in depressive symptoms (ß = -0.40; *P*<0.001). Higher age had a positive (Odds Ratio = 2.04, 95% CI: 1.21–3.43; *P*<0.01) and type 2 diabetes had a negative effect on the meaningful reduction of depressive symptoms (Odds Ratio = 0.12, 95% CI: 0.04–0.35; *P*<0.001).

**Conclusions:**

The reduction of diabetes distress is a statistical predictor of improvement of depressive symptoms. Diabetes patients with comorbid depressive symptomatology might benefit from treatments to reduce diabetes-related distress.

## Introduction

Depression is more prevalent in people with diabetes than in people without the condition [1–2] and is associated with various adverse effects on diabetes-related and general health outcomes. Not only major depression but also elevated depressive symptoms were found to be associated with reduced self-care [3], a greater number of diabetes complications [4], higher health-care costs [5], poorer quality of life [6] and elevated mortality [7] in people with diabetes.

Therefore, screening and intervention for depression is recommended in several guidelines [8–9]. Meta-analyses could also demonstrate that psychosocial interventions in people with diabetes and depression showed favorable effects on depression status [10–11].

However, knowledge about demographic, clinical or psychosocial factors facilitating treatment response in people with diabetes and depression is sparse [11]. Depression in diabetes is often associated with non-modifiable risk factors such as younger age, female gender, type 2 diabetes, presence of complications or longer diabetes duration [12–14] as well as modifiable risk factors such as glycemic control or diabetes-related distress [12,15].

Especially the relationship between depression and diabetes-related distress is of interest, given the substantial correlations between the two conditions. In cross-sectional analyses, moderate-to-strong associations ranging from *r* = 0.44 up to *r* = 0.56 [15–19] were found.

A better understanding of non-modifiable and modifiable factors that impede or facilitate improvement of depression would be helpful to optimize treatment options for depression in diabetes and to identify people with diabetes who are likely to experience improvement of depressive symptoms or who are in need of extended treatment support to achieve such improvement, respectively.

To get a better understanding of factors related to the remission of depression, we performed a secondary analysis of a previously published randomized controlled trial in which the impact of a cognitive behavioral approach for the treatment of subthreshold depression was tested.

In this secondary analysis, we pooled participant data regardless of the randomized group allocation and performed multivariate analyses to identify demographic and clinical factors potentially related to an improvement of depressive symptoms. The role of a reduction of diabetes-related distress for the improvement of depressive symptoms was of special interest in this analysis.

## Materials and methods

### Study sample

This analysis included data from the DIAMOS intervention study (identifier NCT01009138), approved by the Ethics Committee of the State Medical Chamber of Baden-Württemberg, Germany, and conducted at a tertiary referral center for diabetes (Diabetes Center Mergentheim), Germany. A detailed description and a report of intervention effects are available elsewhere [20] and are not focused in this secondary analysis, thus, treatment group affiliation was only used as a control variable. There was a 12-month period between baseline and follow-up measurements.

### Measures

Diabetes-related distress was assessed using the German version of the Problem Areas In Diabetes (PAID) scale [21]. It consists of 20 items describing problems related to living with diabetes. Respondents rate the severity of each problem on a 5-point Likert scale (0 –‘not a problem’ to 4 –‘serious problem’). Summed item scores are multiplied by 1.25, resulting in a total score ranging from 0 to 100. The German version of the PAID was attested good psychometric properties [15].

Depressive symptoms were assessed using the German version of the Center for Epidemiologic Studies Depression (CES-D) scale [22]. Respondents rate the frequency of the occurrence of depressive symptoms during the last week on a four-point Likert scale (0 –‘rarely or never’ to 3 –‘most of the time’). Total scores range from 0 to 60, with higher scores indicating increased intensity of depressive symptoms. The German version of the CES-D has high reliability and validity [22].

Glycemic control was operationalized by the HbA1c value. Venous blood samples were collected at the time of psychometric assessments; all samples were analyzed in the center’s laboratory using high-performance liquid chromatography (HPLC) performed with the Bio-Rad Variant II Turbo analyzer. Normal laboratory values range from 23.5 to 43.2 mmol/mol [4.3–6.1%].

Socio-demographic and diabetes-related patient characteristics were gained from electronic patient records.

### Statistical analyses

Statistical analyses were performed using SYSTAT 10.2 (Systat Software, Point Richmond, California, USA). Descriptive statistics, Student’s *t*-tests and multivariate linear and logistic regression analyses were used. *P*-values of <0.05 were considered to indicate statistical significance in all the analyses.

As questionnaire scores for both CES-D and PAID were positively-skewed, square root transformation was performed on the baseline and 12-month follow-up scores of the CES-D and the PAID total scores to avoid heteroskedasticity. These transformed variables were used in all analyses.

The concept of reliable change [23–24] was used to analyze whether a statistically meaningful reduction of diabetes-related distress can predict a statistically meaningful reduction of depressive symptoms. Using square root transformed baseline scores of the CES-D and the PAID scales, a reliable change index of 1.36 (15.43 in raw scores) was calculated for the PAID (based on baseline Cronbach’s *α* = 0.91 and standard deviation *(SD)* = 1.63) and an index of 1.01 (9.53 in raw scores) was calculated for the CES-D (based on baseline Cronbach’s α = 0.82 and *SD* = 0.86). Thus, a reduction of more than 1.36 points in the square root transformed PAID total score and more than 1.01 points in the equally transformed CES-D total score between the baseline measurement and the 12-month follow-up was considered to reflect a reliable change in diabetes-related distress or depressive symptoms, respectively.

A binary logistic regression model was used to analyze whether known demographic and clinical risk factors for depression or reliable change of diabetes-related distress can predict reliable change in the severity of depressive symptoms. Therefore, reliable change in diabetes-related distress (vs. no reliable change in diabetes-related distress), age, female gender (vs. male gender), type 2 diabetes (vs. type 1), diabetes duration, glycemic control, presence of late diabetes complications (vs. no complications) were used as the independent variables and reliable change in depressive symptoms (vs. no reliable change in depressive symptoms) was used as the dependent variable in the logistic regression model.

To account for the dichotomization of the outcome measures, a multiple linear regression analysis was also performed. Risk factors for depression (age, gender, diabetes type, diabetes duration, glycemic control and presence of long-term complications) and changes in diabetes-related distress between baseline and follow-up were used as the independent variables; depressive symptoms (CES-D) at the 12-month follow-up were used as the dependent variable, adjusted for baseline depressive symptoms.

All regression models were adjusted for the treatment group affiliation. Variables for age, diabetes duration and HbA1c scores were z-transformed (Mean = 0, *SD* = 1) in order to facilitate interpretability of their results in the logistic regression model. Odds ratios for these variables are based on the respective standard deviation.

## Results

### Sample characteristics

181 people with diabetes completed the study (age 45.0±13.6 years, 57% female, 63% type 1 diabetes, 14.5±10.7 years of diabetes duration, 52% with late complications, HbA1c 73±5 mmol/mol). The sample characteristics are displayed in Table 1. People with a reliable reduction of diabetes-related distress showed higher depressive symptoms and diabetes-related distress at baseline and follow-up. They were also less often afflicted with one or more late complications of diabetes (41% vs. 58%, *P* = 0.027) than people without a reliable reduction of diabetes-related distress (Table 1).

**Table 1:** Baseline characteristics and group differences.

	Total	RC of DRD	No RC of DRD	*P*^a^
*N*	181	63	118	
Age (years)	45.0 ± *13*.*6*	46.0± *13*.*8*	46.1 ± *13*.*4*	0.138
Female gender	*103* (57)	*37* (59)	*66* (56)	0.717
Type 1 Diabetes	*114* (63)	*41* (65)	*73* (62)	0.670
Diabetes duration (years)	14.5 ± *10*.*7*	12.6 ± *10*.*0*	15.6 ± *11*.*0*	0.067
Late complications (yes/no)	*95* (52)	*26* (41)	*69* (58)	0.027
HbA1c (mmol/mol)	73 ± *5*	73 ± *2*	75 ± *6*	0.812
HbA1c (%)	8.8 ± *1*.*7*	(8.7 ± *2*.*0*)	(8.8 ± *1*.*6*)	
Baseline depressive symptoms				
Raw	23.3 ± *8*.*1*	25.7 ± *8*.*3*	22.0 ± *7*.*7*	0.004
Square-root transformed	4.7 ± *0*.*9*	5.0 ± *0*.*8*	4.6 ± *0*.*8*	0.003
Follow-up depressive symptoms				
Raw	18.2 ± *11*.*0*	13.8 ± *8*.*7*	20.5 ± *11*.*4*	<0.001
Square-root transformed	4.0 ± *1*.*4*	3.5 ± *1*.*2*	4.3 ± *1*.*4*	<0.001
Change in depressive symptoms				
Raw	5.1 ± *11*.*8*	12.0 ± *11*.*0*	1.5 ± *10*.*5*	<0.001
Square-root transformed	0.7 ± *1*.*4*	1.5 ± *1*.*4*	0.3 ± *1*.*3*	<0.001
Baseline diabetes-related distress				
Raw	39.5 ± *18*.*4*	46.6 ± *16*.*4*	35.8 ± *18*.*4*	<0.001
Square-root transformed	6.1 ± *1*.*6*	6.7 ± *1*.*3*	5.7 ± *1*.*7*	<0.001
Follow-up diabetes-related distress				
Raw	30.8 ± *19*.*2*	19.4 ± *12*.*3*	36.9 ± *19*.*5*	<0.001
Square-root transformed	5.2 ± *1*.*9*	4.1 ±*1*.*6*	5.8 ± *1*.*7*	<0.001
Change in diabetes-related distress				
Raw	8.7 ± *18*.*4*	27.2 ± *10*.*8*	-1.2 ± *13*.*4*	<0.001
Square-root transformed	0.8 ± *1*.*7*	2.6 ± *1*.*1*	-0.1 ± *1*.*2*	<0.001

Data are *n* (%) or M ± *SD*.

^a^Two-tailed significance of differences between people with and without reliable change in diabetes-related distress (Student’s t-test or Pearson’s χ^2^-test).

RC = Reliable change; DRD = Diabetes-related distress

21.5% of the population showed a reliable change in both depressive symptoms and diabetes-related distress, while 47.5% did not show a reliable reduction in either. 17.7% only showed reliably reduced depressive symptoms and 13.3% only showed reliably reduced diabetes distress.

A moderate correlation was found between change scores of depressive symptoms and change scores of diabetes-related distress (*r* = 0.46; *P*<0.001) (S1 Fig).

### Factors associated with improvement of depressive symptoms

**Fig 1:**
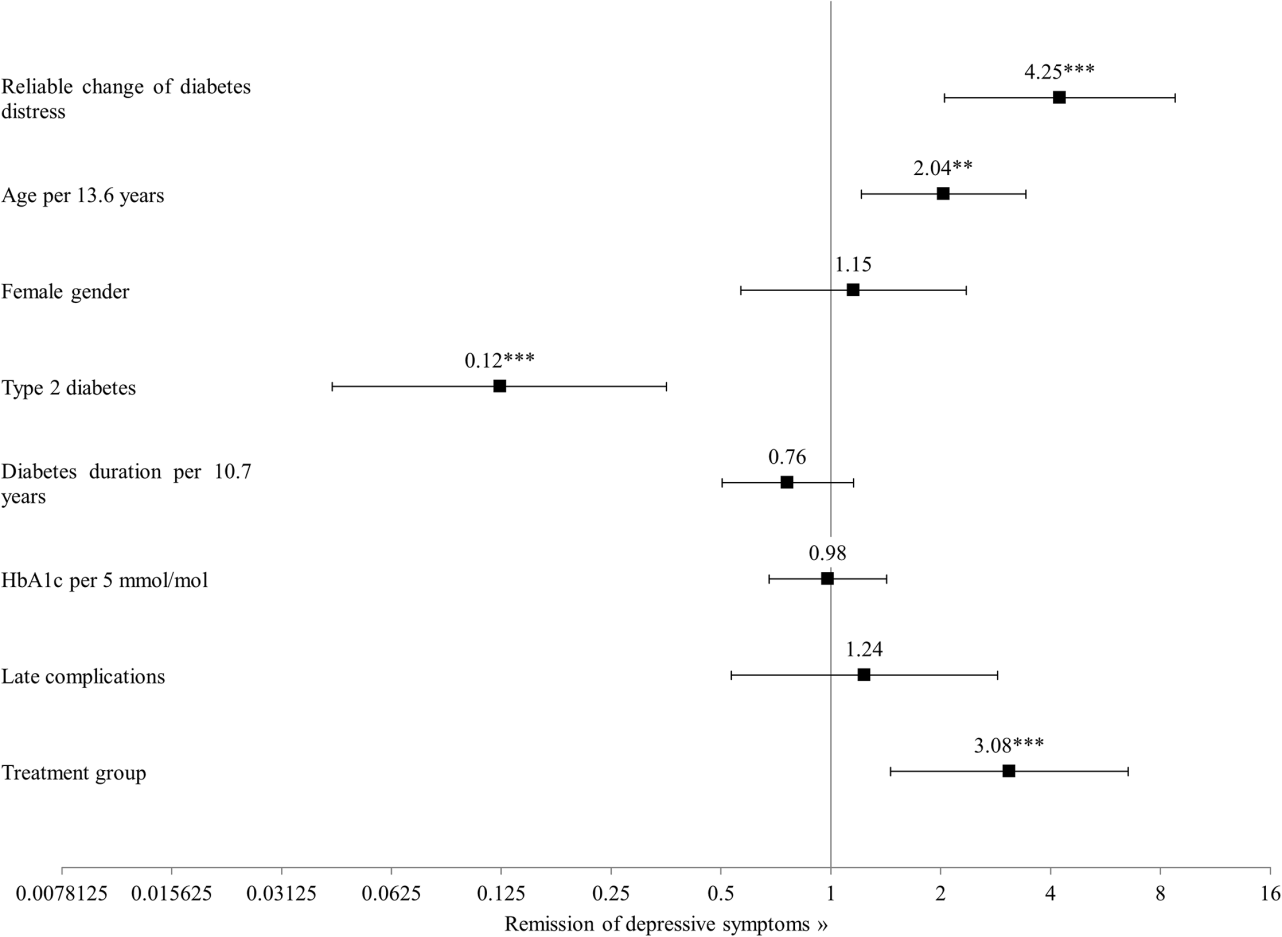
Odds ratios for the reliable reduction of depressive symptoms. **P<0.01; ***P<0.001.

The logistic regression model (Fig 1) showed that people with a reliable reduction of diabetes distress were four times as likely to show reliable improvement of depressive symptoms (Odds Ratio = 4.25, 95% CI: 2.05–8.79; *P*<0.001) (Fig 1). An increase in age by 13.6 years doubled the odds of having a reliable reduction of depressive symptoms (Odds Ratio = 2.04, 95% CI: 1.21–3.43; *P*<0.01). Having type 2 diabetes strongly reduced these odds (Odds Ratio = 0.12, 95% CI: 0.04–0.35; *P*<0.001).

In the multiple linear regression model (R^2^ = 0.22, Standard Error = 1.20), improvement of diabetes-related distress was significantly associated with reduced depressive symptoms (ß = -0.40, *P*<0.001). Thus, diabetes distress was found to predict improved depressive symptoms independently from cut-off scores. None of the other included risk factors were predictive of reduced depressive symptoms in the linear regression analysis (Table 2).

**Table 2:** Linear regression analysis of depressive symptoms at 12-months after baseline.

	ß	SE	t	*P*
Change in diabetes-related distress	-0.40	0.05	-5.79	<0.001
Age	-0.12	0.13	-1.27	0.205
Sex	0.04	0.19	0.62	0.540
Diabetes type	-0.17	0.25	-1.88	0.062
Diabetes duration	0.06	0.11	0.72	0.470
HbA1c	0.00	0.10	0.03	0.975
Late complications	0.04	0.22	0.55	0.583
Baseline depressive symptoms	0.35	0.11	4.99	<0.001
Treatment group	-0.08	0.19	-1.12	0.264

SE = Standard Error

## Discussion

In this prospective study, we analyzed the associations of known risk factors with improved depressive mood, particularly focusing on the role of diabetes-related distress. Our results show that a statistically meaningful reduction of diabetes-related distress was significantly associated with a statistically meaningful reduction of depressive symptoms. This suggests that improved diabetes-related distress is an important independent factor for the improvement of depressed mood in people with diabetes, as people with reduced emotional burden related to diabetes and its treatment were more likely to experience improvement of depressive symptoms. This finding was also confirmed in analyses using continuous measures of change in depressive symptoms and diabetes-related distress.

The relationship between depression and diabetes distress is a focus of current research. Recent longitudinal analyses by Burns, Deschênes and Schmitz [25] as well as Ehrmann, Kulzer, Haak and Hermanns [26] showed that diabetes distress was prospectively associated with depressive symptoms. Ehrmann et al. found that elevated diabetes distress increased the future risk of incident elevated depressive symptoms 2.5-fold. In people with elevated depressive symptoms and diabetes distress, the risk of persistent elevated depressive symptoms was increased by a factor of 3.3, confirming the results of an earlier analysis by Pibernik-Okanovic et al. [27].

Based on the strong cross-sectional and longitudinal associations between diabetes-related distress and depression, a reduction of diabetes distress was expected to be associated with reduced depressive symptoms. However, previous evidence was primarily based on observational and epidemiological studies in which the severity of diabetes distress was not manipulated. This analysis bridges a gap by using interventional data to examine how a reduction of diabetes-related distress affected the improvement of depressive symptoms. Our finding is also meaningful from a practical perspective, as the reduction of diabetes-related distress can be enhanced primarily through diabetological interventions such as self-management education [28–29] or treatment modification (e.g., simplification of diabetes treatment, more effective diabetes treatment) [30].

Results on other risk factors showed a positive effect of older age and a negative effect of type 2 diabetes on the reliable reduction of depressive symptoms. The positive effect of older age was expected, as psychological resilience against depression is known to increase with age [31–32].

The negative effect of having type 2 diabetes could be partially explained by findings that depression later in life is associated with increased somatic burden [33] and that people with type 2 diabetes tend to be older and more impaired by medical comorbidities. However, both age and the long-term complications of diabetes were controlled for in the analysis. Furthermore, neither the body mass index nor the frequency of blood glucose testing, both known to be associated with non-remission of depression in people with type 2 diabetes [34], had a significant effect when added to the model (not reported). Our results suggest that depressive symptoms might be more persistent in people with type 2 diabetes. Hence, more intensive and longer-term psychological care may be advisable for this group.

Somewhat surprisingly, given that women are more likely to be depressed [35], gender had no effect on the dependent variable in our analysis, suggesting that the reduction of depressive symptoms is independent of gender.

We observed no effects of diabetes duration, glycemic control or long-term diabetes complications on the improvement of depressive symptoms.

Our results should be considered in the light of the limitations of this post-hoc analysis. The study lacked sufficient power for the conducted analysis and for the use of reliable change as a criterion for improved depressive symptoms and diabetes distress. This resulted in relatively small subgroups, which limited the statistical power. Second, the percentage of people with type 2 diabetes was relatively low, limiting the generalizability of our results for this group. The low number of people with type 2 diabetes was due to the specific tertiary care setting in which the study was conducted. On the other hand, our analysis may provide some balance, as studies that include people with type 1 diabetes tend to be rarer. Third, the concept of reliable change reduces the likelihood that people with a lower baseline score will show statistically reliable improvement at all. Also, regression to the mean has to be considered as a factor contributing to changes in depressive symptoms and diabetes distress.

The strengths of the study lie in the analysis of interventional data to close an important gap in the understanding of the relationship between diabetes distress and depressive symptoms as well as in the use of both dichotomized and continuous measures of change to operationalize a meaningful reduction of diabetes-related distress and depressive symptoms.

In summary, the study showed that reduced diabetes-related distress can predict improved depressive symptoms in people with diabetes. This result corroborates epidemiological and observational evidence and emphasizes the importance of addressing diabetes-specific issues in the treatment of people with diabetes and depressive mood. Appropriate screening tools such as the PAID or the Diabetes Distress Scale [36] are available for use to identify depressed people with concomitant elevated diabetes distress [37]. In contrast to depression and its symptoms, diabetes-specific emotional burden can be addressed using low-threshold interventions such as self-management education [28–29], more specific interventions such as DIAMOS, or available web-based approaches developed by Nobis et al. [38] or Rondags, de Wit, Twisk and Snoek [39]. Investigating the causal relationship between diabetes-related distress and depressive symptoms needs further research as the development of diabetes-specific psychological interventions can benefit from understanding the sources of depressive symptoms.

## Supporting information

S1 Data Microsoft Excel 2010 data file. (XLSX)

S1 Fig Correlation of change in CES-D and PAID with cut-lines for reliable change. A = Reliable change in diabetes-related distress; B = Reliable change in depressive symptoms and diabetes-related distress; C = Reliable change in depressive symptoms; D = No reliable change.
(PDF)
