# Supplementary figures and images for: Reduction of diabetes-related distress predicts improved depressive symptoms: A secondary analysis of the DIAMOS study
Source: PLoS One. 2017 Jul 10;12(7):e0181218. doi: 10.1371/journal.pone.0181218 (PMC5507326; doi:10.1371/journal.pone.0181218)

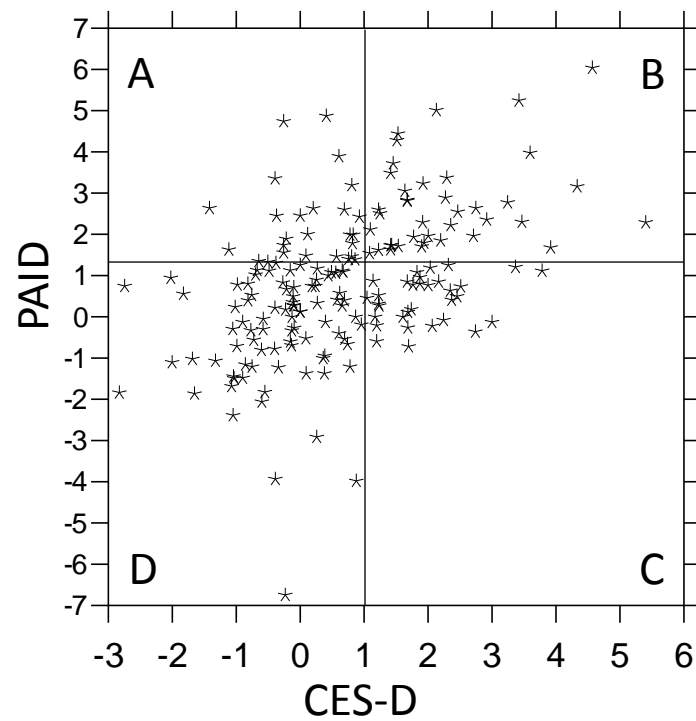

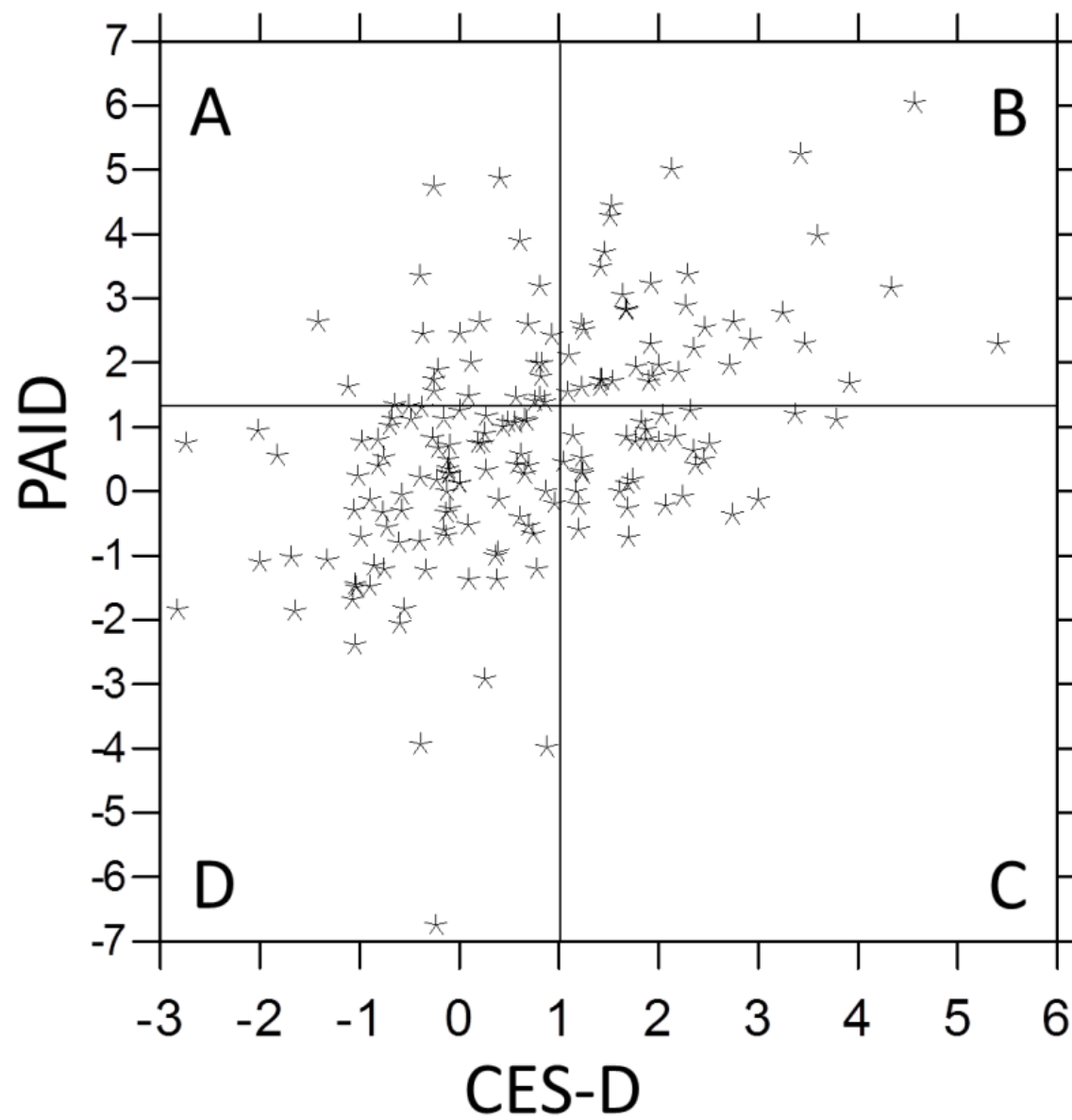

Supplement: S1 Fig — A = Reliable change in diabetes-related distress; B = Reliable change in depressive symptoms and diabetes-related distress; C = Reliable change in depressive symptoms; D = No reliable change. (PDF) [file pone.0181218.s002.pdf]
